# An acoustofluidic device for the automated separation of platelet-reduced plasma from whole blood

**DOI:** 10.1038/s41378-024-00707-3

**Published:** 2024-06-24

**Authors:** Zhehan Ma, Jianping Xia, Neil Upreti, Emeraghi David, Joseph Rufo, Yuyang Gu, Kaichun Yang, Shujie Yang, Xiangchen Xu, Jean Kwun, Eileen Chambers, Tony Jun Huang

**Affiliations:** 1 Thomas Lord Department of Mechanical Engineering and Materials Science, Duke University, Durham, NC USA; 2 Department of Biomedical Engineering, Duke University, Durham, NC USA; 3 Department of Pediatrics, Duke University, Durham, NC USA; 4 Duke Transplant Center, Department of Surgery, Duke University School of Medicine, Durham, NC USA

**Keywords:** Engineering, Physics

## Abstract

Separating plasma from whole blood is an important sample processing technique required for fundamental biomedical research, medical diagnostics, and therapeutic applications. Traditional protocols for plasma isolation require multiple centrifugation steps or multiunit microfluidic processing to sequentially remove large red blood cells (RBCs) and white blood cells (WBCs), followed by the removal of small platelets. Here, we present an acoustofluidic platform capable of efficiently removing RBCs, WBCs, and platelets from whole blood in a single step. By leveraging differences in the acoustic impedances of fluids, our device generates significantly greater forces on suspended particles than conventional microfluidic approaches, enabling the removal of both large blood cells and smaller platelets in a single unit. As a result, undiluted human whole blood can be processed by our device to remove both blood cells and platelets (>90%) at low voltages (25 Vpp). The ability to successfully remove blood cells and platelets from plasma without altering the properties of the proteins and antibodies present creates numerous potential applications for our platform in biomedical research, as well as plasma-based diagnostics and therapeutics. Furthermore, the microfluidic nature of our device offers advantages such as portability, cost efficiency, and the ability to process small-volume samples.

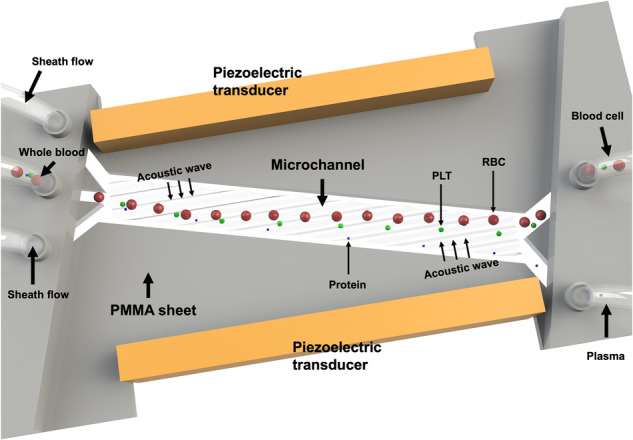

## Introduction

Platelet-reduced plasma^1–3^ (also known as platelet-poor plasma) is essential for many therapeutic approaches and is used as a diagnostic sample in clinical medicine. Platelet-reduced plasma is an aqueous substance that contains numerous analytes, including proteins such as antibodies, lipids, extracellular vesicles (EVs), metabolites, and cell-free nucleic acids^1–3^. Cellular components, including white blood cells (WBCs), red blood cells (RBCs), and platelets, in whole blood should be completely removed to accurately detect these analytes and prevent potential contamination during diagnostic^4–6^ or therapeutic procedures. In addition, platelet-reduced plasma also plays essential roles in coagulation tests^7,8^, platelet aggregation tests^9^, and wound healing promotion in plastic surgery^10–12^. Currently, centrifugation is still the “gold-standard” method for plasma separation^13^ because it can generate platelet-reduced plasma from whole blood in an efficient and cost-effective manner. However, this process is labor-intensive, as multiple centrifugation steps are typically needed, and the high centrifugal forces generated have been shown to activate and fragment platelets, leading to altered EV profiles and cell-free nucleic acid profiles^14^. As a result, the development of automated plasma isolation technologies that minimize sample handling and reduce the number of processing steps is urgently needed to help standardize plasma-based biomarker research and reduce the confounding variables introduced by current sample processing protocols.

Researchers have developed various lab-on-a-chip techniques for automated plasma separation from whole blood, including sedimentation^15,16^, microfiltration^17,18^, hydrodynamic-based separation^19^, dielectrophoresis^20^, and acoustofluidics^21–30^, to address these shortcomings. However, these methods focus solely on removing blood cells from the plasma, neglecting the influence of platelets on downstream applications. Among these approaches, acoustofluidics utilizes acoustic radiation force and/or acoustic streaming for cell manipulation^31–35^ in a contact-free manner, making it capable of efficiently removing blood cells with high biocompatibility^36–39^. Due to these advantages, acoustofluidic techniques have strong potential for different biomedical applications^23,40–44^. Regarding blood cell separation, current acoustofluidic separation methods require separate processing units to isolate larger WBCs and RBCs, followed by the removal of smaller platelets because the acoustic radiation force exerted on platelets (diameters: 2–3 μm) is 20 times less than the acoustic radiation force applied to blood cells (diameters: 5-10 μm). If the acoustic radiation force for platelet isolation increases, blood cells will experience excessive forces that trap them inside the microfluidic channel and block the channel^45^. Additionally, excessive forces may damage the cell structure and/or alter cell properties^46–48^.

This work introduces an impedance mismatch-assisted, tilted-angle acoustofluidic separation technology with a removal rate greater than 90% for cellular components and platelets. Tilted-angle pressure nodes generated by piezoelectric transducers inside a plastic channel provide the necessary acoustic radiation force to achieve cell isolation. These nodes occur when the acoustic transducer and resonance frequency match and a standing acoustic wave propagates through the lateral poly(methyl methacrylate) (PMMA) channel wall into the microchannel. We achieved platelet removal from plasma by carefully tuning the acoustic impedance force introduced and using an acoustic impedance mismatch method. Our acoustofluidic device isolates platelets from plasma with a WBC/RBC/platelet removal rate of 85–95% and a throughput of 20 μl/min. Overall, with the advantages of high biocompatibility, affordability, and automated processing, the newly developed acoustofluidic-based plasma separation method shows promise in facilitating the standardization of plasma-based diagnostics. Furthermore, because current centrifugal devices are size-prohibitive for neonates and infants^49^, our device could eventually provide life-saving platelet-free plasma for treating a variety of early childhood medical conditions, such as neonatal sepsis^50^, hemolytic disease of the newborn^51^, thrombocytopenia^52^, and congenital coagulation factor deficiencies^53^.

## Results

### Working mechanism

**Fig. 1:**
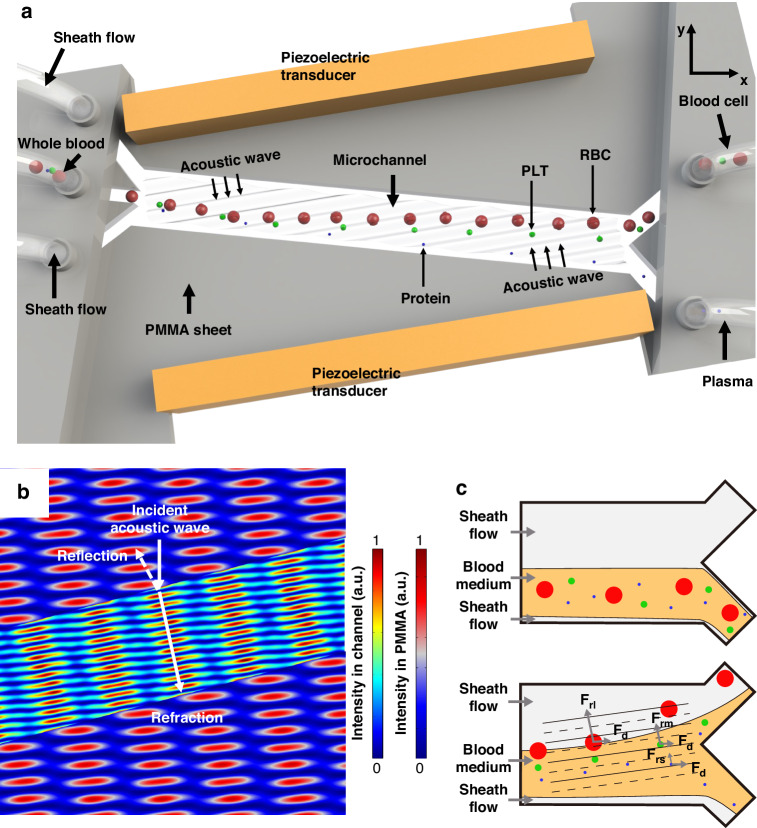
Schematic of impedance mismatch-assisted, tilted-angle acoustofluidic plasma separation **a** Working principle of the impedance mismatch-assisted acoustofluidic device. A pair of piezoelectric transducers are attached to the lateral side of the chip. **b** Results of the acoustic field distribution simulation within the microchannel. Multiple parallel pressure nodes are arranged inside the microchannel when filled with homogeneous fluid. **c** Schematic of the acoustofluidic separation process. White blood cells (WBCs), red blood cells (RBCs), platelets, and plasma proteins flow in the blood medium toward the lower outlet when no acoustic signal is applied. When an acoustic signal is applied, the WBCs and RBCs experience a large acoustic radiation force and are pushed toward the upper outlet. The relatively large platelets experience a greater acoustic radiation force than drag force, causing them to flow toward the upper outlet. Most of the smaller plasma proteins remain in the blood medium and flow toward the lower outlet, indicating successful plasma separation

The impedance mismatch-assisted, tilted-angle acoustofluidic plasma separation device consists of a two-layer structure and a pair of piezoelectric transducers. The external layers of the structure are composed of PMMA sheets fabricated through computer numerical control (CNC) machining. Figure 1a displays the 3D diagram and schematic of the device. Initially, the microchannel is engraved on the bottom of the platform and covered by the top layer. A pair of piezoelectric transducers are then bonded parallel to the lateral grooves using epoxy. The detailed constitution of the whole device is included in the Supplementary Information (Fig. S1). Separation is achieved by vibrating the piezoelectric transducers, which generate a standing acoustic wave inside the microchannel, forming multiple acoustic pressure nodes. The device is placed inside a large petri dish of ice water during the separation process to counteract the rapid heading of the piezoelectric transducers. Figure 1b displays the simulation result for an array of tilted-angle pressure nodes, which form when the microchannel is filled with a homogeneous medium. Due to the difference in the acoustic speed between PMMA and phosphate-buffered saline *(*PBS), refraction occurs at the liquid‒solid interface, contributing to the tilted-angle acoustic pressure distribution field. The piezoelectric transducers have a resonance frequency of 7.0 MHz. Our design allows multiple pressure nodes and anti-pressure nodes to form in parallel at the center of the microchannel. Upon entering the microchannel, blood cells are subjected to varying acoustic radiation forces depending on size differences, as described in the equations below. Since RBCs and WBCs (6–17 µm) are larger than platelets (2–3 µm), they are subject to greater acoustic radiation forces.

1 $${F}_{{\rm{R}}}=-\left(\frac{k{p}_{0}^{2}{V}_{c}{\beta }_{m}}{4}\right)\varPhi \left(\beta ,\rho \right)\sin \left(2{kx}\right)$$

2 $$\varPhi \left(\beta ,\rho \right)=\frac{5{\rho }_{c}-2{\rho }_{m}}{2{\rho }_{c}+{\rho }_{m}}-\frac{{\beta }_{c}}{{\beta }_{m}}$$

Equation 1 shows the relationships among the properties of the cell, the acoustic parameters of pressure and wavenumber, the vertical distance from the pressure, and the acoustic radiation force. ${F}_{{\rm{R}}}$ indicates the acoustic radiation force, ${p}_{0}$ and $k$ are symbols for the acoustic pressure and wavenumber, respectively, ${V}_{c}$ denotes the cell volume, $\varPhi$ represents the acoustic contrast factor, and $x$ is the vertical distance from the pressure. Equation 2 defines the acoustic contrast factor $\varPhi$, which is a function of the density and compressibility of the cell (${\rho }_{c}$ and ${\beta }_{c}$) in relation to the ${\rho }_{m}$ and ${\beta }_{m}$ of the media.

As the blood and PBS flow through the channel, blood components are subjected to the acoustic radiation force defined in Eq. 1. The volume predominantly determines the variation in the acoustic radiation force and the corresponding particle trajectories. Additionally, the relationship between the particle and medium density and compressibility contributes to the separation efficiency. In the bulk acoustic wave (BAW)-dominant mode, large blood cells can be removed under the effect of acoustic radiation forces; however, platelets are more difficult to remove due to their smaller size. Additionally, due to its density, human whole blood forms inhomogeneous fluids with conventional sheath fluid (such as 0.15 M NaCl and PBS). A mismatch in the acoustic impedances between whole blood and sheath flow was introduced to address this issue, promoting the separation of platelets due to the addition of an acoustic impedance force. The introduced acoustic impedance force creates an additional driving force. When a strong acoustic standing wave is applied, this difference in acoustic impedance drives a portion of the blood medium toward the other outlet, assisting in the removal of platelets. In a heterogeneous medium consisting of high and low impedance phases, a liquid with high impedance tends to move toward the acoustic pressure node. This phenomenon is known as liquid relocation in a half-wavelength channel containing only one pressure node. In a microchannel with multiple pressure nodes, this phenomenon causes the high-impedance sample flow to be pushed toward the other outlet (Fig. 1c). The impedance mismatch introduces the concept of the acoustic impedance force^54,55^, which is expressed in the following equation:

3 $${f}_{{im}}=-\frac{1}{4}{\left|{p}_{1}\right|}^{2}\nabla \frac{\rho }{{z}^{2}}-\frac{1}{4}{\left|{\nu }_{1}\right|}^{2}\nabla \rho$$

In Eq. 3, ${p}_{1}$ and ${\nu }_{1}$ represent the acoustic pressure and acoustic velocity in the inhomogeneous fluid, respectively, *z* represents the fluid acoustic impendence, and $\rho$ represents the density of the inhomogeneous fluid, where *z* and $\rho$ are variables that change with coordinates. This equation illustrates that the acoustic impedance force is a gradient force dependent on the density (*ρ*) and impedance (*z* = *ρc*), achieving its maximum value at the interface between sheath flow and blood flow, where the acoustic impedance differs in our devices. Here, c represents the sound velocity. The direction of the acoustic impendence force points from the high-density/impendence medium to the low-density/impendence medium on the interface of the sheath fluid and blood. Figure S2 additionally illustrates the simulated acoustic impedance force within the channel. Under the acoustic impendence force, blood sample flow will obtain momentum toward the blood cell/platelet outlet. The blood flow momentum generates a drag force^56,57^, which is directly proportional to the diameter of the bioparticles. This force disproportionately affects larger blood cells and platelets, exerting a significantly greater influence on them than on antibodies and other nanoscale bioparticles. Therefore, the acoustic impedance force acts as another driving force, which allows the efficient manipulation of bioparticles ranging from 2 to 3 micrometers in size, becoming a helpful supplement to the acoustic radiation force in our acoustofluidic devices.

### Characterization of the acoustofluidic device via particle separation

**Fig. 2:**
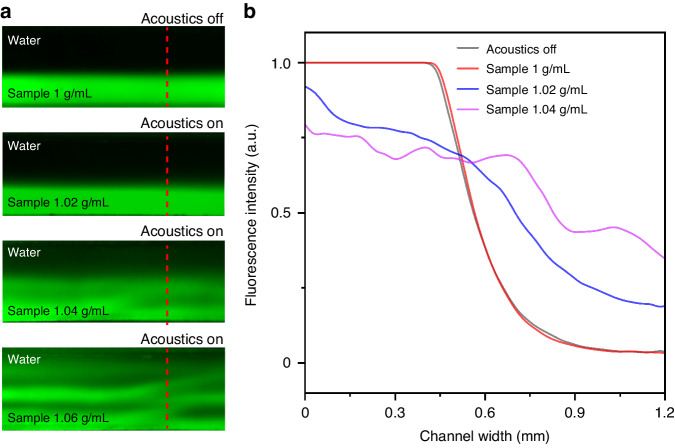
Influence of the driving force on separation performance due to the acoustic impedance **a** Images showing the motion of the PBS fluorescent solution with a density difference with DI water before and after the acoustic signal was applied. **b** Intensity level showing the separation effect caused by the acoustic impedance force. The *x*-axis represents the position along the dashed lines in **a** from the bottom of the channel to the top. The strongest intensity correlates to 1.0 on the scale

Several samples of 10× PBS were diluted with double-distilled water (ddH_2_O) to densities of 1.0, 1.02, and 1.04 g/mL and used as the sample flow to determine the effect of the acoustic impedance force on the inhomogeneous medium. Fluorochrome (46960, Sigma, USA) was added to visualize the sample flow. As shown in Fig. 2a, when the sample flow density was 1.0 g/mL (the same as that of ddH_2_O), the sample flow was not influenced by the acoustic field due to the homogeneity of the medium. This observation reveals that the drag force from acoustic streaming and the acoustic radiation force induced by standing acoustic waves do not impact the fluid. However, when the sample density was gradually increased to 1.02 g/mL and 1.04 g/mL, significant sample flow movements were observed when the acoustic field was active. Figure 2b further displays the normalized fluorescence intensity across the dashed lines in Fig. 2a. This result shows that the sample flow obtains extra momentum from the acoustics due to the impedance difference resulting from the density difference. Thus, the platelets will experience Stoke’s drag force as the impedance mismatch forces the sample to flow toward the waste outlet, yielding a platelet-reduced plasma.

Prior to whole blood separation, a particle separation experiment was conducted to validate the effects of the acoustic radiation force and the drag force enhanced by acoustic impedance differences. According to the formula used to calculate the acoustic radiation force, particles with larger volumes experience greater force and shift longer distances along the cross-section of the channel, entering the outlet on the other side. The acoustic effect on particles of different sizes was evaluated by testing 5 μm and 400 nm yellow‒green polystyrene particles purchased from Magsphere. Two hundred microliters of fluorescent polystyrene particles were diluted with 40 ml of ddH_2_O to a final concentration of 0.5%. Pure ddH_2_O was selected for sheath flow. The calculated acoustic impedances of the sample flow and sheath flow did not differ significantly. Particle trajectory was monitored using a microscope.

**Fig. 3:**
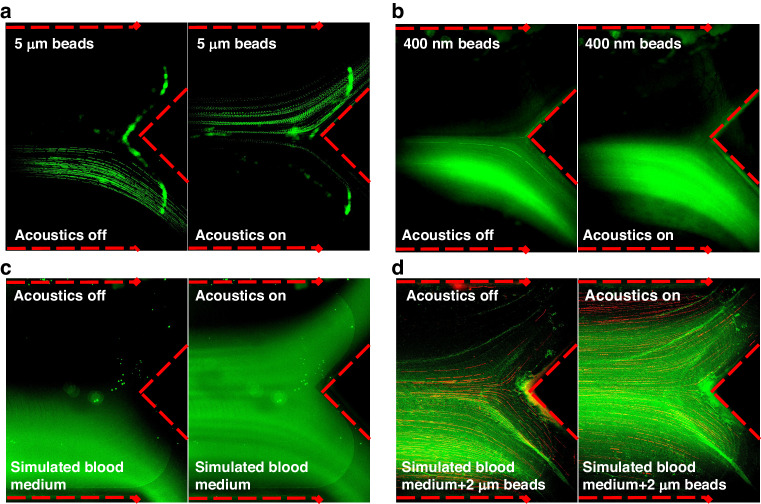
Characterization of fluorescent particle separation using a mismatch-assisted, tilted-angle acoustofluidic separation device **a** Stacked image showing the traced path of 5 μm fluorescent polystyrene particles before and after applying the acoustic signal. **b** Stacked image showing the traced path of 400 nm fluorescent polystyrene particles before and after applying the acoustic signal. **c** Image showing the motion of the simulated blood medium labeled with green fluorescent dye before and after the acoustic signal was applied. **d** Image showing the motion of the simulated blood medium labeled with green fluorescent dye, as well as 2 μm red fluorescent polystyrene particles, before and after the acoustic signal was applied

Figure 3a shows that 5 μm polystyrene particles flow close to the lower channel wall when no acoustic signal is applied. The flow is deflected toward the upper outlet after the function generator is turned on, showing the dominant effect of the acoustic radiation force on 5 μm particles. In contrast, Fig. 3b shows that when the particle size decreased to 400 nm, the influence of the acoustic radiation force is almost nonexistent. The particles continue to flow toward the same outlet, indicating the negligible effect of the acoustic radiation force. Comparing the results for particles of different sizes, the acoustic radiation force significantly affects targets larger than 5 µm in homogeneous fluid but has a much weaker influence on nanoscale particles. A particle-free medium whose impedance was adjusted to simulate human whole blood was used to evaluate the effect of the acoustic impedance force on blood sample flow and to validate that a heterogeneous fluid with obvious acoustic impedance differences in different portions would be disturbed within an acoustic field. A total of 4.5 ml of Percoll density gradient medium (P1644, Sigma Aldrich, USA) was mixed with 0.5 ml of 1.5 M NaCl and 5.686 ml of 0.15 M NaCl to simulate blood with a density of 1.06 g/ml. Then, 25 μl of 0.01% fluorescein sodium was added as a fluorescent label. Pure ddH_2_O was once again used for sheath flow. The microchannel was then observed under a fluorescence microscope. Figure 3c displays part of the high-impedance sheath flow diffusing to the lower outlet when the acoustic wave is transmitted into the microchannel, successfully showing the acoustic impedance-assisted liquid relocation. Figure 3d shows that 2 μm polystyrene particles can be manipulated toward the upper outlet, along with the simulated blood medium in Fig. 3c, indicating that the acoustic impedance force enables the separation of smaller particles.

### Acoustofluidic plasma separation

The efficiency of WBC/RBC and platelet removal is an essential indicator for blood separation techniques. The flow rate and driving voltage applied to the acoustic transducer are two significant factors that influence the acoustofluidic plasma separation performance. The device was held in a petri dish filled with ice water to continually cool the entire system and prevent the generation of air bubbles in the channel. The WBC/RBC/platelet removal rate was quantified as a function of the flow rate and driving voltage. Based on the premise of equal flow rates for the upper outlet and bottom outlet, the removal rate $\varphi$ is defined as the ratio of WBCs/RBCs/platelets collected from the blood cell outlet and the sum of WBC/RBC/platelet outflow in both outlets as follows:

4 $$\varphi =\frac{{c}_{b}}{{c}_{b}+{c}_{p}}$$

**Fig. 4:**
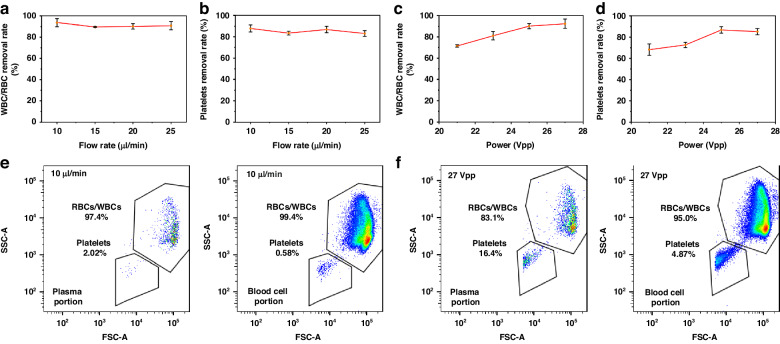
RBC/WBC/platelet removal rates of the acoustofluidic device as a function of the driving voltage applied to the acoustic transducer and sample flow rate **a**, **b** The relationship between the WBC/RBC/platelet removal rates and the sample flow rate. **c**, **d** The influence of the driving voltage on the removal rates of WBCs/RBCs and platelets, as characterized *via* flow cytometry. **e**, **f** Separation performance of blood samples with a flow rate of 10 μl/min and an applied voltage of 27 Vpp, based on flow speed and acoustic power tests, respectively

In Eq. 4, ${c}_{b}$ and ${c}_{p}$ represent the WBC/RBC/platelet concentrations in the blood cell and plasma portions, respectively. All the results were obtained by processing the flow cytometry data. Due to the negligible change in flow rate compared with the faster sheath flow (200 μl/min), we expected no difference in WBC/RBC separation. In Fig. 4a, b, the voltage was fixed at 25 Vpp, and the flow rate was increased from 10 to 25 μl/min; the WBC/RBC removal rate fluctuated between 87 and 97%, while the platelet removal rate fluctuated between 80 and 91%. However, when the driving voltage was increased from 21 to 27 Vpp and the sample flow was maintained at 20 µl/min, the WBC/RBC and platelet removal rates improved significantly from 73 to 97% and 63 to 90%, respectively (Fig. 4c, d). When the applied voltage was increased, a greater acoustic radiation force acted upon the WBCs/RBCs; similarly, the platelets were subjected to greater acoustic radiation and acoustic streaming forces. Consequently, the displacement of all the blood cells and platelets increased, improving the WBC/RBC and platelet removal rates. The flow cytometry profiles of blood samples separated at 10 μl/min during the flow rate test and those separated at 27 Vpp during the acoustic power test are shown separately in Fig. 4e, f, indicating successful blood cell removal. The platelet removal rate decreased slightly when the input voltage increased from 25 Vpp to 27 Vpp, revealing the perturbation caused by stronger acoustic streaming when too much power was applied. When operated at 20 μl/min and 25 Vpp, the impedance mismatch-assisted, tilted-angle acoustofluidic device could reach over 90% for both the WBC/RBC and platelet removal rates while maintaining relatively high throughput. Under these operating conditions, the power consumption of the chip was recorded at 3.45 watts. This measurement was obtained using a Tektronix TCP2020 Current Probe, which has a current of 138 mA.

### Analysis of separated blood components

**Fig. 5:**
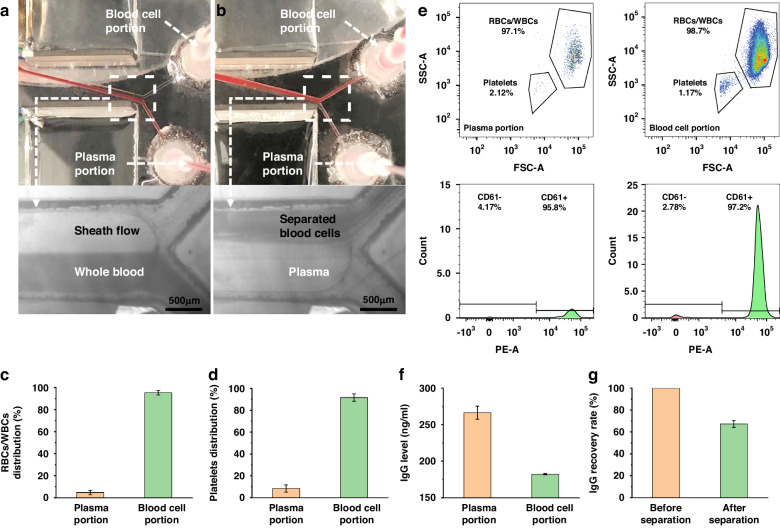
Blood component analysis of separated human whole blood **a** Whole blood is collected at the upper outlet when the acoustic transducer is turned off. **b** When the acoustic transducer is turned on, blood cells are separated to the lower outlet. The separation efficiency of (**c**) blood cells and **d** platelets. **e** Flow cytometry results for the collected blood cell and plasma portions. After separation, the remaining platelets were labeled with PE-conjugated anti-CD61 antibodies. **f** ELISA was used to determine the IgG distribution in the blood cell and plasma portions. **g** IgG recovery rate when comparing the plasma portion with original human whole blood using ELISA

After setting the driving voltage to 25 Vpp and the sample flow rate to 20 μl/min, the blood plasma was again separated for hemocyte and antibody analyses. The upper and lower sheath flow rates were 200 μl/min and 20 μl/min, respectively. We meticulously balanced the outflows from both outlets to ensure that they had identical flow rates. Consequently, with the sample flow (*F*_s_) set at 20 μl/min, the dilution factor (*D*) is calculated as *D* = *F*_s_: (*F*_up_ + *F*_down_ + *F*_s_)/2. This calculation resulted in a dilution ratio of 1:6. During the plasma separation process, as shown in Fig. 5a, b, turning the transducer off caused the whole blood to flow to the lower outlet, and the sheath flowed to the upper outlet. After the transducer was turned on, the blood cells were separated and directed to the upper outlet. Figure 5c, d display the separation efficiency of WBCs/RBCs and platelets following the flow cytometry analysis. With removal rates of WBCs/RBCs and platelets greater than 90%, a small number of RBCs and platelets remained in the separated plasma. A detailed flow cytometry profile is shown in Fig. 5e. When comparing the plasma and blood cell portions, significantly more WBCs/RBCs and platelets were present in the latter. This observation indicated that the majority of WBCs/RBCs and platelets were distributed in the blood cell portion. According to forward scatter (FSC) and side scatter (SSC) levels, all events could be divided into two groups representing blood cells and platelets. A CD61 fluorescence test showed that most of the components of the platelet group were intact. The high percentage of CD61-positive cells in both the blood cell and platelet fractions showed that the acoustofluidic separation procedure produced little to no harm to blood cells. An enzyme-linked immunosorbent assay (ELISA) was performed on the preprocessed original whole blood and processed samples, including isolated blood cells and plasma, to further evaluate the sample mixing and characterize the retention rate of plasma antibodies. The distribution and recovery rate of IgG were then calculated by comparing the normalized IgG concentration of these samples determined using ELISA. The IgG distribution and recovery rate are shown in Fig. 5f, and g, respectively, indicating that more than 60% of the human IgG remained in the plasma medium. Nevertheless, the noticeable migration of some antibodies toward alternative outlets was attributed to the instability of the acoustic streaming. This instability was prompted by variations in the acoustic field distribution, particularly at its initiation and termination points. We could employ a gradient channel in the future to mitigate the effects of acoustic streaming and enhance the IgG recovery rate. According to previous work^55^, this method could stabilize the acoustic streaming within the channel. These antibody results indicated that the impedance mismatch-assisted, tilted-angle acoustofluidic device performs well when removing larger blood cells from plasma, providing an ideal fluid experiment for downstream analysis or diagnosis.

## Discussion

Platelet-reduced plasma, due to its lack of clotting factors and enrichment of fibrinogen, plays an important role in coagulation tests^7,8^ and platelet aggregation tests^9^ clinically and has an encouraging effect on promoting tissue adhesion during wound healing^10,11^, which provides enormous potential for its application in plastic surgery^12^. In this study, we reported an impedance mismatch-assisted, tilted-angle acoustofluidic separation method capable of separating platelet-reduced plasma from whole blood. This method enables label-free separation of cellular and subcellular particles, including those smaller than RBCs, from biofluids (such as blood) for research and diagnostic applications. Our acoustofluidic device isolates blood plasma with an 85-95% WBC/RBC/platelet removal rate and a throughput of 20 μl/min. By tuning the driving voltages and sample flow rate within a small range (21–27 Vpp, 10–25 μl/min), we can effectively control the separation efficiency of blood plasma. In contrast with traditional acoustofluidic methods, the purposeful introduction of acoustic impedance mismatching in our technique provides an additional external force (acoustic impedance force) to manipulate smaller particles (such as platelets).

Another important factor that influences separation efficiency when conducting experiments is preventing the attenuation of acoustic waves transmitted into the microchannel due to bonding epoxy and the PMMA material. The thickness of the bonding epoxy between the piezoelectric transducers and the PMMA structure greatly influences the intensity of the acoustic field within the channel, as most of the attenuation occurs at this interface. A thin layer (<50 µm) of epoxy will reduce the acoustic attenuation while maintaining the bonding strength between the acoustic transducers and the PMMA chip.

Our experiment takes advantage of inhomogeneous fluids by introducing the acoustic impedance force as an extension of the acoustic radiation force separation. The introduced acoustic impedance force can manipulate bioparticles ranging from 2 to 3 micrometers in the bulk wave frequency range (<10 MHz). Acoustic impedance force can be achieved by regulating the density difference between whole blood and sheath fluids in this acoustofluidic method. Introducing this additional physical phenomenon represents an increased understanding in the field of acoustofluidics, in general, and has the potential to be used in other applications, such as drug delivery and tissue engineering.

The cost of production is always a major consideration regarding the application of certain industrial research. The fabrication of this acoustofluidic separation device requires CNC machining to process the chip body, which can be further optimized for more cost-efficient fabrication. Laser cutting has been widely used in PMMA machining as a relatively low-cost alternative with high accuracy and has proven to be a reliable method^58–60^. Given that the material of this device is irrelevant to either separation efficiency or blood cell viability, any nontoxic solid material can replace PMMA to reduce the cost or when seeking alternatives due to a shortage of PMMA. Polycarbonate^61^, polystyrene^62^, and other plastic materials can serve as suitable bases. In general, variable processing techniques and sufficient types of optional materials broaden the potential application of our acoustofluidic separation device.

In summary, we developed a bulk wave acoustofluidic platform that utilizes the acoustic radiation force from tilted-angle pressure nodes and the acoustic impedance force introduced by acoustic impedance mismatch to achieve small-scale separation. The ability of the platform to separate platelet-reduced plasma surpasses that of conventional bulk wave separation methods in the manipulation of bioparticles ranging from 2-3 micrometers. In addition to isolating platelet-reduced plasma from whole blood with high efficiency, our acoustofluidic device possesses several advantages, including high biocompatibility, the label-free nature, and continuity of the separation process. With these advantages, it has significant potential in applications relating to novel apheresis devices in animal models and diagnostics for vulnerable populations such as infants and small children.

## Materials and methods

### Device fabrication

Two layers of PMMA sheets and 5 adapters are required to assemble the acoustofluidic device. The two PMMA layers were machined by a computer numerical control cutting machine. A microchannel with a width of 1.27 mm and a depth of 0.5 mm was engraved on the bottom of the PMMA sheet. The 5 adapters connected the inlets and outlets to the tubing. Piezoelectric plates (SMPL20W15T1R111) were purchased from STEMINC, Inc., and those with good performance were screened based on their S11 curves (Fig. S3). A thin layer of epoxy was applied on the side of the device to attach the piezoelectric transducers to the lateral surface. The thickness of the epoxy layer was minimized to avoid the attenuation of acoustic transmission at the interface while still maintaining the bonding strength.

### Numerical simulation

A simulation was utilized to optimize the device design and increase our understanding of the acoustic pressure distribution in the microchannel of the acoustofluidic plasma separation device. Specifically, a numerical simulation was conducted using a finite-element-based software package, COMSOL Multiphysics. The “Pressure Acoustics” module was used to solve the acoustic field of the device within the transducer-functioning area by applying incident acoustic waves at 7.0 MHz, the resonance frequency of the transducer applied in the experiment. The simulation results showed parallel pressure nodes forming an array in the center of the microchannel, with a tilted angle to the channel wall.

### Acoustofluidic system setup

The blood sample and sheath fluid were injected into the acoustofluidic plasma separation device through a four-channel peristaltic pump controlled by a computer. The acoustofluidic plasma separation device was placed in a petri dish filled with ice water to prevent overheating and bubble formation. The water level was above the microchannel and below the upper surface of the PMMA sheet. An RF signal generator (AFG 3011, Tektronic, USA) and a power amplifier (25A250A, Amplifier Research, USA) provided coherent AC signals to the acoustic transducer. An impedance matcher connected the power amplifier and the acoustofluidic device to tune the acoustic impedance and minimize power loss. After being bonded to the fluidic chamber, the resonance frequency of the acoustic transducer was measured using a vector network analyzer (VNA 2180, Array Solutions, USA). The sample flow rate and acoustic power were tuned by adjusting the peristaltic pump and signal generator.

### Blood samples and buffer solution

Human whole blood samples, purchased from Zenbio, Inc., were prepared with PBS to maintain blood cell viability and establish the necessary acoustic impedance difference for separation. The flow rates of the sheath flow were fixed at 20 μl/min and 200 μl/min. Pre- and postacoustic separation samples were collected and processed: 2 µl of processed sample was mixed with 2 µl of PE-labeled anti-CD61 antibody (Bioligand), incubated for 1 min, and diluted with 1 ml of PBS. The unprocessed original sample was diluted further by a factor of 10 compared with processed samples by mixing a 1 µl sample with 1 µl PE-labeled anti-CD61 antibody and 5 ml PBS. Red blood cells and platelets were quantified using a flow cytometer (BD FACSCanto II, BD Biosciences, USA). The flow rate of the cytometer was fixed, and the collection time was set to 2 min. The collected data were processed with FlowJo V10 (BD Biosciences, USA).

### Plasma IgG analysis

ELISA was performed to quantify the protein levels in the separated samples and evaluate the separation efficiency. Human IgG was chosen as the representative biomarker due to its abundance in human whole blood. A human IgG ELISA kit (88-50550, Thermo Fisher, USA) was used to measure IgG levels in the samples before and after separation. The plate was read with a microplate reader (B-SHT, BioTek, USA). Based on this information, a standard curve was drawn using a four-parameter logistic curve calculator. The absorbance of different samples was substituted into the curve to obtain the IgG concentration.

### Supplementary information

Supplementary Information
