# Supplementary material for: An acoustofluidic device for the automated separation of platelet-reduced plasma from whole blood
Source: Microsyst Nanoeng. 2024 Jun 24;10:83. doi: 10.1038/s41378-024-00707-3 (PMC11194281; doi:10.1038/s41378-024-00707-3)
Supplement: Supplementary file 1 — Supplementary Information [file 41378_2024_707_MOESM1_ESM.docx]

Supplementary material

**An Acoustofluidic Device for the Automated Separation of Platelet-reduced Plasma from Whole Blood**

Zhehan Ma,^1^ ^†^ Jianping Xia,^1^ ^†^* Neil Upreti,^2^ Emeraghi David,^3^ Joseph Rufo,^1^ Yuyang Gu,^1^ Kaichun Yang,^1^ Shujie Yang,^1^ Xiangchen Xu,^1^ Jean Kwun,^4^ Eileen Chambers,^3^ and Tony Jun Huang^1^*

^1^ Thomas Lord Department of Mechanical Engineering and Materials Science, Duke University, Durham, North Carolina, 27708, USA.

^2^ Department of Biomedical Engineering, Duke University, Durham, North Carolina, 27708, USA.

^3^ Department of Pediatrics, Duke University, Durham, North Carolina, 27708, USA.

^4^ Duke Transplant Center, Department of Surgery, Duke University School of Medicine, Durham, North Carolina, 27708, USA.

^†^ These authors contributed equally to this work

Corresponding author: [jianping.xia@duke.edu](mailto:jianping.xia@duke.edu), [tony.huang@duke.edu](mailto:tony.huang@duke.edu)


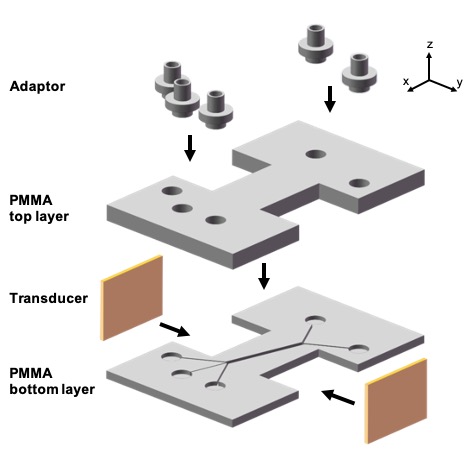


**Figure S1.** An exploded 3D diagram illustrates the structure of the acoustofluidic platelet separation device, which is comprised of two layers of polymethyl methacrylate (PMMA). These layers are meticulously assembled using a hot press machine, ensuring a precise and durable construction. The device features intricately CNC-machined microfluidic channels, inlets, and outlets, all carefully carved into the PMMA material. For a comprehensive overview of the device’s fabrication process, detailed information is provided in the methods section.

**
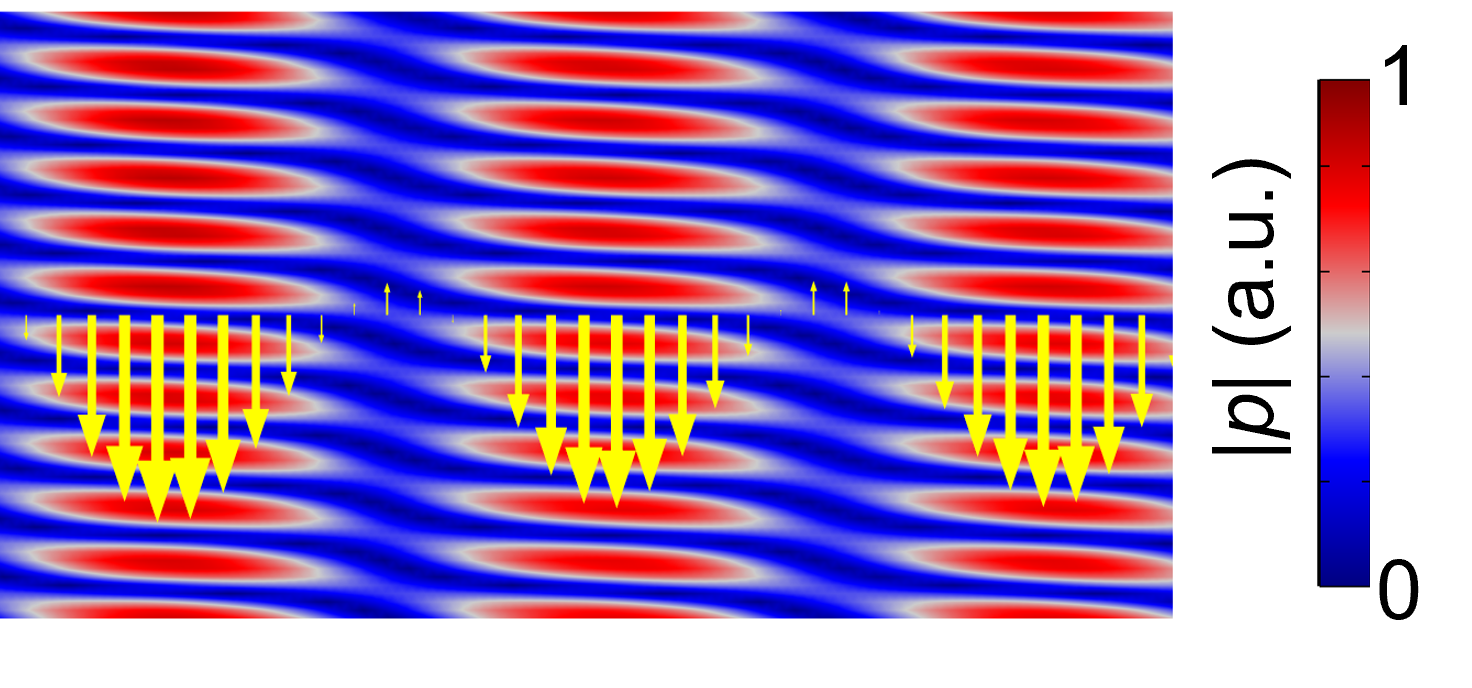
**

**Figure S2.** The distribution of acoustic pressure amplitude and the direction of impedance forces (indicated by yellow arrows) within the channel created by a pair of transducers. The impedance force, which arises from the gradient in fluid density, is present exclusively at the interface between the blood sample and the sheath flow. The diagram identifies the top fluid as a high-density fluid (1060 kg/m^3^) and the bottom fluid as a low-density fluid (1000 kg/m^3^), illustrating how the acoustic impedance force directs the sample towards the sheath flow for enhanced cell separation.


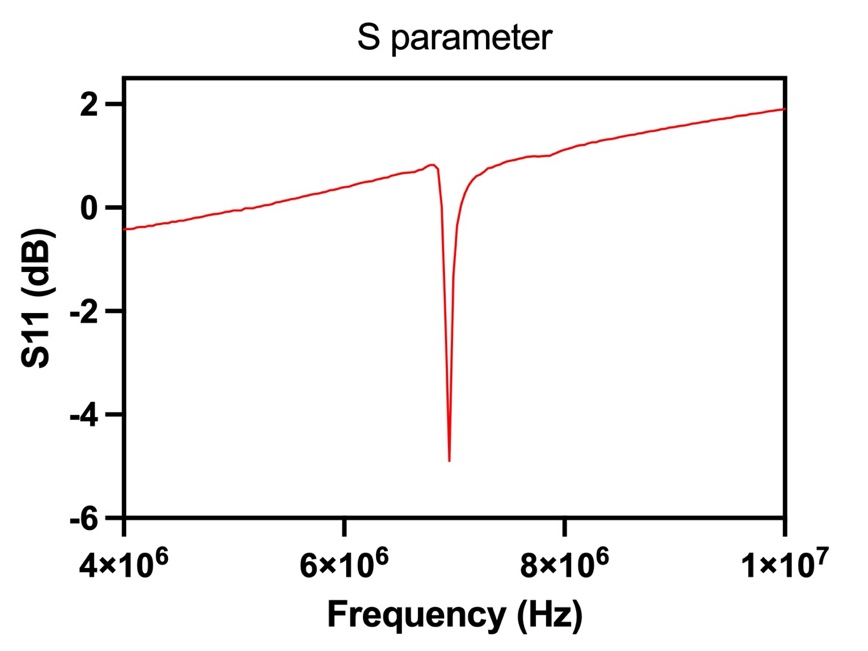


**Figure S3.** S11 curves of the piezoelectric transducers attached to the acoustofluidic platelet separation device.
